# Monitoring arthropods under the scope of the LIFE-BEETLES project: I - Baseline data with implementation of the Index of Biotic Integrity

**DOI:** 10.3897/BDJ.12.e124799

**Published:** 2024-07-23

**Authors:** Sébastien Lhoumeau, Noelline Tsafack, Sónia Manso, Telma Figueiredo, Abrão Leite, Laurine Parmentier, Maria Teresa Ferreira, Paulo A. V. Borges

**Affiliations:** 1 University of the Azores, cE3c- Centre for Ecology, Evolution and Environmental Changes/Azorean Biodiversity Group, CHANGE – Global Change and Sustainability Institute, School of Agricultural and Environmental Sciences, Rua Capitão João d´Ávila, Pico da Urze, 9700-042, Angra do Heroísmo, Azores, Portugal University of the Azores, cE3c- Centre for Ecology, Evolution and Environmental Changes/Azorean Biodiversity Group, CHANGE – Global Change and Sustainability Institute, School of Agricultural and Environmental Sciences, Rua Capitão João d´Ávila, Pico da Urze, 9700-042 Angra do Heroísmo, Azores Portugal; 2 Institut Méditerranéen de Biodiversité et d’Ecologie, Avignon Université, CNRS, IRD, Aix Marseille Université, 84911, Avignon, France Institut Méditerranéen de Biodiversité et d’Ecologie, Avignon Université, CNRS, IRD, Aix Marseille Université, 84911 Avignon France; 3 Regional Secretariat of Environment and Climate Change, Project LIFE BEETLES (LIFE 18NAT/PT/000864), Rua do Galo n118, 9700-040, Angra do Heroísmo, Azores, Portugal Regional Secretariat of Environment and Climate Change, Project LIFE BEETLES (LIFE 18NAT/PT/000864), Rua do Galo n118, 9700-040 Angra do Heroísmo, Azores Portugal; 4 Rua Fernando Pessoa, nº99 R/C DTO 2765-483, Estoril, Portugal Rua Fernando Pessoa, nº99 R/C DTO 2765-483 Estoril Portugal; 5 Rua da Oliveira nº8, 9700-136 Sé, Angra do Heroísmo, Portugal Rua da Oliveira nº8, 9700-136 Sé Angra do Heroísmo Portugal; 6 IUCN SSC Atlantic Islands Invertebrate Specialist Group, Angra do Heroísmo, Azores, Portugal IUCN SSC Atlantic Islands Invertebrate Specialist Group Angra do Heroísmo, Azores Portugal; 7 IUCN SSC Species Monitoring Specialist Group, Angra do Heroísmo, Azores, Portugal IUCN SSC Species Monitoring Specialist Group Angra do Heroísmo, Azores Portugal

**Keywords:** biodiversity, conservation, habitat quality, island, Azores, Index of Biotic Integrity (IBI) framework

## Abstract

**Background:**

The urgent need for conservation efforts in response to the global biodiversity crisis is exemplified by initiatives, such as the EU LIFE BEETLES project. This project aims to preserve endangered arthropod species that are crucial for ecosystem functionality, with a focus on endemic beetle species in Flores, Pico and Terceira Islands (Azores, Portugal): *Tarphiusfloresensis* Borges & Serrano, 2017, *Pseudanchomenusaptinoides* (Tarnier, 1860) and *Trechusterrabravensis* Borges, Serrano & Amorim, 2004. These species are single island endemics respectively from Flores, Pico and Terceira. They are threatened by environmental degradation, facing the dual challenge of restricted distribution and habitat degradation due to the spread of invasive plants.

The project aims to enhance habitat quality and biodiversity conservation through habitat restoration and plant invasive species control measures. These measures are funded by the European Commission and coordinated by the Azorean Environment Directorate-General. The current Data Paper evaluates the effectiveness of the LIFE BEETLES project in improving habitat quality and offers insights into the balance between habitat restoration efforts and endangered species conservation in island ecosystems, utilising as ecological indicator the Index of Biotic Integrity (IBI) framework.

**New information:**

This study establishes a comprehensive database derived from a long-term arthropod monitoring survey that used SLAM (Sea, Land and Air Malaise) traps and pitfall traps. Our findings present a proxy for assessing the overall habitat quality for endemic invertebrates, using arthropods as main indicators.

From September 2020 to June 2023, a total of 31 SLAM traps were monitored. The traps were set up as follows: seven in Flores (three in mixed forest and four in native forest), 10 in Pico (four in mixed forest and six in native forest) and 14 in Terceira (three in mixed forest and 11 in native forest). Traps were monitored every three months.

In addition, we surveyed the epigean fauna in 19 transects with 15 non-attractive pitfall traps per transect. The transects were set up during two weeks at the end of August every year between 2020 and 2023. Eight transects were established in Flores, consisting of one in pasture, four in mixed forest and three in native forest. Six transects were established in Pico, consisting of two in pastures and four in native forest. Five transects were established in Terceira, consisting of two in mixed forest and three in native forest.

A total of 243 arthropod taxa were recorded, with 207 identified at the species or subspecies level. These taxa belonged to four classes, 24 orders and 101 families. Out of the 207 identified taxa, 46 were endemic, 60 were native non-endemic, 80 were introduced and 21 were of indeterminate status. Habitat information is also provided, including general habitat and dominant species composition. This publication contributes to the conservation of highly threatened endemic beetles by assessing habitat quality, based on arthropod communities and habitat description (e.g. native or exotic vegetation).

Using the Index of Biotic Integrity (IBI) to comparing pre- and post-intervention data, we found no significant change within the epigean community. In contrast, the understorey community sampled with SLAM traps experienced a slight global decrease in biotic integrity over the study period. These findings suggest that the short duration of the study may not be sufficient to detect significant changes, as ecosystem recovery often requires long-term monitoring. The observed changes in the understorey community may be attributed to disturbances from intervention activities, highlighting the need for ongoing monitoring to assess long-term ecological resilience and recovery.

## Introduction

The global biodiversity crisis is intensifying (Singh 2002, Shivanna 2020), with critical species loss and subsequent erosion of vital ecosystem services. This trend has triggered the launch and implementation of conservation projects on a global scale (Mammola et al. 2020, Parks et al. 2022). In response to the need to protect biodiversity, these projects aim to mitigate the loss of biodiversity and the associated services delivered by these diverse ecosystems.

Within the broader biodiversity crisis, arthropods are critical to ecosystem functionality and stability (Mishra and Omkar 2023). Arthropods, which include insects and spiders, play a critical role in a wide range of ecological processes (Losey and Vaughan 2006). They are important as pollinators, decomposers and predators and are essential for maintaining the balance of ecosystems, influencing aspects ranging from plant reproduction to nutrient cycling (Chapman et al. 2013). Given the intricate connections between arthropods and ecosystem services, it seems crucial to develop conservation strategies that target this taxon.

The LIFE BEETLES project (Bringing Environmental and Ecological Threats Lower to Endangered Species) is one such initiative dedicated to enhancing the population size, distribution area and conservation status of three endemic beetle species: *Tarphiusfloresensis* Borges & Serrano, 2017 (Coleoptera, Zopheridae), *Pseudanchomenusaptinoides* (Tarnier, 1860) (Coleoptera, Carabidae) and *Trechusterrabravensis* Borges, Serrano & Amorim, 2004 (Coleoptera, Carabidae). These single-island endemics are threatened by environmental degradation, facing the dual challenge of restricted distribution and habitat degradation, largely due to the spread of invasive alien species (Borges et al. 2020). The LIFE BEETLES project is a five-year initiative (January 2020 - December 2024) launched by the European Commission and coordinated by the Azorean Environment Directorate-General (LIFE Units - E.3. and E.4.). Its aim is to conserve the abovementioned species of endemic beetles that are not protected by the Habitats Directive. These species were assessed as Endangered (*T.terrabravensis*) or Critically Endangered (*P.aptinoides*, *T.floresensis*) on the 2017 IUCN Red Lists (Borges et al. 2017). Similar threats affect all species, as they are highly dependent on good quality habitats.

The project's operational objectives focused on increasing the availability of habitat for the target species, both in terms of quantity and quality, with the aim of reversing the observed decline in their populations. The project aimed to restore native habitats by increasing the density of trees and shrubs to promote shadowing, humidity and higher soil cover with ferns and bryophytes. Additionally, it aimed to prevent, control and limit the spread of vascular plants known to be Invasive Alien Species (IAS) and promote native ferns through active dispersal of spores.

To ensure operational monitoring of the project and achieve these goals, a new scientific index has been developed. The LIFE BEETLES project has adopted the Index of Biotic Integrity (IBI) framework for assessing the biological integrity of arthropod communities in the context of islands. This framework was informed by the previous work of the Biodiversity of Arthropods of Laurisilva of the Azores (BALA) project (Borges et al. 2005, Gaspar et al. 2008, Gaspar et al. 2011).

The IBI is a multimetric tool that integrates several key components of arthropod communities to provide quick insights into the quality of forest sites. It was originally conceptualised by Cardoso et al. (2007) with a primary focus on the epigean arthropod community. Recently, Tsafack et al. (2023b) have made enhancements to this index. The expansion focuses on two aspects: the IBI-SLAM, which concentrates on the understorey arthropod community and the IBI-canopy, which emphasises the canopy arthropod community. Tsafack and collaborators' publication (Tsafack et al. 2023b) exhaustively outlines the detailed parameters for computation. This synthetic index has been designed for applied conservation actions as it emerges as a comprehensive and user-friendly tool. The IBI framework has been previously successful in assessing the effectiveness of conservation interventions in island contexts (Tsafack et al. 2023a).

In this study, the IBI framework is strategically applied to evaluate the LIFE BEETLES project's contributions to the sustainability of endemic beetle species populations and overall conservation efforts. The research aims to provide a nuanced understanding of the project's impact. Previous research has highlighted the effectiveness of the IBI in measuring the quality of forest habitats (Cardoso et al. 2007, Tsafack et al. 2023a, Tsafack et al. 2023b). However, its specific use in measuring the success of conservation projects in island ecosystems, particularly in the context of threatened beetle species, has not been extensively researched. Rigorous evaluations of the consequences of conservation interventions on habitat quality are still critically needed. This study aims to fill this gap by extending the application of the IBI framework to assess the improvements in habitat quality resulting from the LIFE BEETLES project interventions.

Specifically, we aim to:

1. Present a comprehensive inventory of terrestrial arthropods sampled in mixed and native forests of three Azorean Islands (Flores, Pico and Terceira) under the scope of the LIFE BEETLES projects.

2. Investigate the changes in habitat quality metrics, as derived from the Index of Biotic Integrity (IBI) (Tsafack et al. 2023b), during the implementation of the LIFE BEETLES project.

By addressing these questions, we aim to contribute to the evolving field of conservation impact assessment and offer practical insights into the balance between habitat restoration efforts and the conservation of endangered beetle species in island ecosystems. This research has the potential to provide information for future conservation strategies, ensuring an effective approach to safeguarding the biodiversity and ecological integrity of these island environments.

## General description

### Purpose

The primary purpose of this publication is to present a comprehensive inventory of terrestrial arthropods sampled in mixed and native forests of three Azorean Islands (Flores, Pico and Terceira) under the scope of the LIFE BEETLES project. The presented data include detailed information on the abundance, diversity and composition of arthropod communities collected during the project's arthropod monitoring survey, utilising SLAM (Sea, Land and Air Malaise) traps and pitfall traps.

### Additional information

In addition to the inventory, this data paper conducts a concise analysis of the collected data using the Index of Biotic Integrity (IBI) framework (Tsafack et al. 2023b). The analysis aims to assess the health and integrity of arthropod communities across various forest strata, providing insights into the overall habitat quality in the intervention areas. This integrated approach not only contributes to the understanding of biodiversity in these Azorean Islands, but also offers valuable information for conservation practitioners, researchers and policy-makers.

## Project description

### Title

The use of arthropods as surrogates of habitat quality within the scope of LIFE - BEETLES project.

### Personnel

The Pitfall and SLAM monitoring protocols were conceived and led by Paulo A.V. Borges.

Fieldwork (site selection and experimental setting): Maria Teresa Ferreira, Sónia Manso, Telma Figueiredo and Paulo A.V. Borges.

Fieldwork (authorisation): Azorean Minister of Environment (Lic 58/2020/DRA; Lic 54/2021/DRAAC; Lic 46/2022/DRAAC; 72/2023/DRAAC) and Azorean Minister of Science and Technology (CCPI 30/2020/DRCT; CCPI 33/2021/DRCTD; CCPI 28/2022/DRCT; CCIR-RAA/2023/28).

Fieldwork (sample collection): Flores (Carolina Teixeira, Luis Cravinho, Telma Figueiredo); Pico (Sónia Silva, Carlos Bettencourt, Lídia Nogueira, Paulo Freitas, Catarina Brasil, Joni Figueiredo & Eduardo Silveira); Terceira (Paulo A. V. Borges, Abrão Leite; Lucas Lamelas-Lopez; Sébastien Lhoumeau).

Parataxonomists: Jonne Bonnet (2020); Magí Ramon Martorell, Sébastien Lhoumeau (2021); Emanuela Cosma, Loïc Navarro, Magdalena Majchrzak, Marco Canino, Valentin Moley (2022); Abrão Leite, Laurine Parmentier (2022-2023).

Taxonomist: Paulo A. V. Borges.

Voucher specimen management: Abrão Leite & Laurine Parmentier.

Database management: Sébastien Lhoumeau & Paulo A. V. Borges.

Darwin Core databases: Sébastien Lhoumeau & Paulo A. V. Borges.

### Funding

Main funding for research and fieldwork was obtained from Secretaria Regional do Ambiente e Alterações Climáticas, Project LIFE BEETLES (LIFE18 NAT/PT/0008647).

Funding for parataxonomists was obtained from EU ERASMUS programme through funding to individual students grants.

Additional funding to obtain SLAM traps was obtained from:

- FCT-UIDB/00329/2020-2024 DOI 10.54499/UIDB/00329/2020 (https://doi.org/10.54499/UIDB/00329/2020) (Thematic Line 1 – integrated ecological assessment of environmental change on biodiversity).
- Azores DRCT Pluriannual Funding (M1.1.A/FUNC.UI&D/010/2021-2024).

Data curation and open access of this manuscript were supported by the project:

- MACRISK-Trait-based prediction of extinction risk and invasiveness for Northern Macaronesian arthropods (FCT-PTDC/BIA-CBI/0625/2021).

## Sampling methods

### Study extent

**Figure 1.**
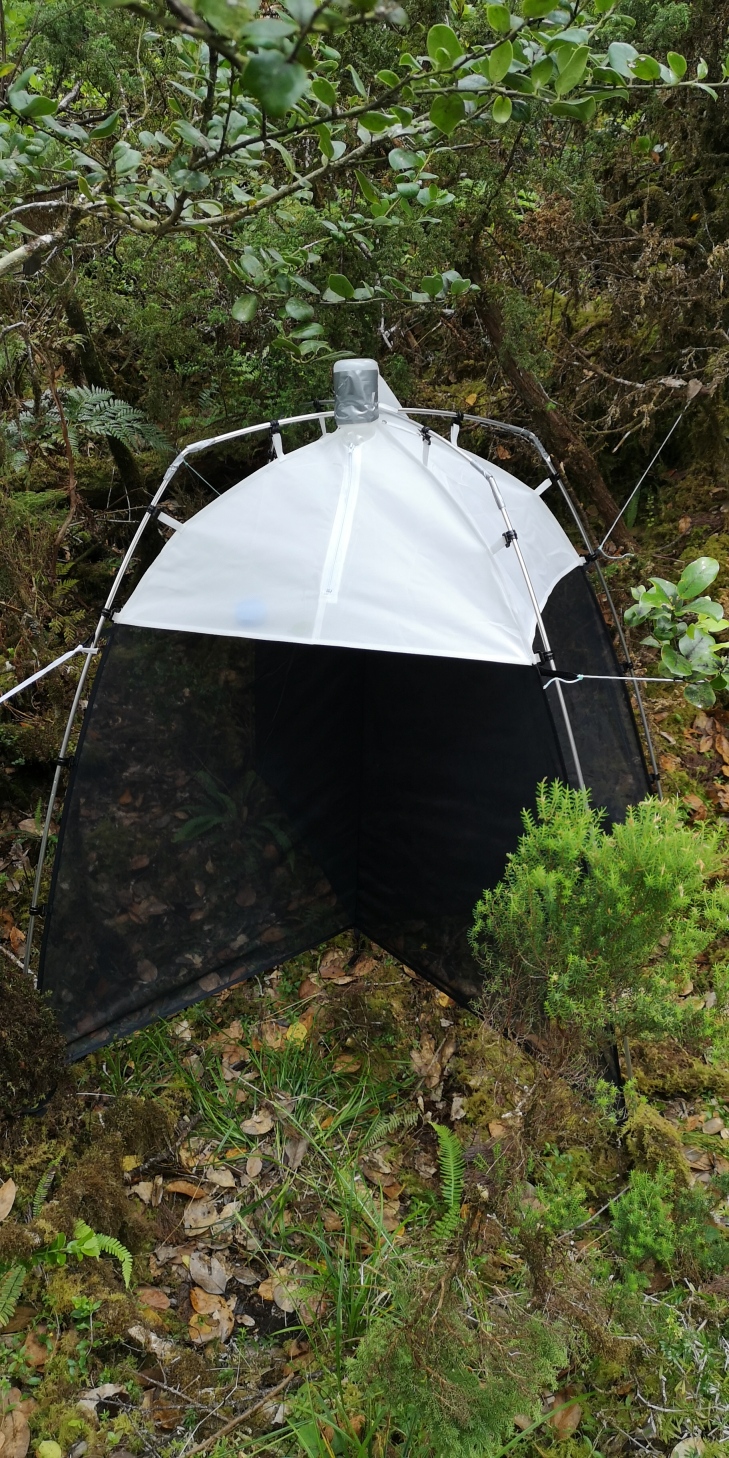
SLAM trap (Sea, Land and Air Malaise trap) (Credit: Paulo A. V. Borges).

From September 2020 to June 2023, a total of 31 SLAM traps (Sea, Land and Air Malaise traps) (Fig. 1) were monitored. The traps were set up as follows: seven in Flores (three in mixed forest and four in native forest), 10 in Pico (four in mixed forest and six in native forest) and 14 in Terceira (three in mixed forest and 11 in native forest). Traps were monitored every three months.

In addition, to evaluate the removal of invasive plants in specific localities, we surveyed the epigean fauna in 19 transects mounting 15 non-attractive pitfall traps in each transect. The transects were set up during two weeks at the end of August every year between 2020 and 2023. Eight transects were established in Flores, consisting of one in natural grassland, four in mixed forest and three in native forest. Six transects were established on Pico, consisting of two in pastures and four in native forest. Five transects were established in Terceira, consisting of two in mixed forest and three in native forest.

### Sampling description

Two types of traps were used.

Passive flight interception SLAM traps (Sea, Land and Air Malaise trap) (Fig. 1) consist of a structure of 110 cm x 110 cm x 110 cm, where the trapped arthropods crawl up the mesh and then fall inside the sampling recipient (Borges et al. 2017). Each one is filled with propylene glycol (pure 1,2-Propanodiol) to kill the captured arthropods and conserve the sample between collections. Although this protocol was developed to sample flying arthropods, by working as an extension of the tree, non-flying species, such as spiders can also crawl into the trap (Borges et al. 2017, Costa and Borges 2021, Lhoumeau et al. 2022), increasing the range of groups that can be sampled by this technique. As a result of this, previous studies have used these traps to analyse diversity and abundance changes in the arthropod communities in Azores pristine forest sites (Matthews et al. 2019, Borges et al. 2020, Lhoumeau and Borges 2023). The samples were collected every 90 consecutive days in most of the sites and, due to logsitical reasons, every 180 consecutive days in one mixed forest from Terceira Island (site TER-PRIBS-T10) between September 2020 and March of 2023.

Additionally, we collected epigean arthropods using pitfall traps for a minimum of two weeks (in some cases, traps were left in the field for one to three days extra due to logistical constraints) during the summers of 2020, 2021, 2022 and 2023. These traps have been shown to effectively sample the epigean arthropod fauna (Borges et al. 2005, Gaspar et al. 2008). The pitfall traps were plastic cups with a top diameter of 42 mm and a depth of 78 mm, placed in the ground so that the lip of the cup was level with the surface. Each transect was equipped with 15 traps spaced 5 m apart. Approximately 60 ml of a non-attractive solution (anti-freeze liquid) with a small proportion of ethylene glycol (10%) and a few drops of liquid detergent filled the traps. The traps were shielded from rain by a white plastic plate fixed to the ground with two pieces of wire, positioned about 5 cm above the surface.

The arthropod samples were then taken to the laboratory and transferred to 96% ethanol.

### Quality control

In the laboratory, standard procedures were followed for specimen sorting and arthropod identification. Species identification was based on somatic and genitalic features and a reference collection was created for all collected specimens, regardless of whether they were identified at the species level. The specimens were identified at the species level by assigning them a morphospecies code number and depositing them at the Dalberto Teixeira Pombo Insect Collection (DTP), University of Azores (Terceira Island). Taxonomic nomenclature and the colonisation status of the species follows the last checklist of Azorean arthropods (Borges et al. 2022).

## Geographic coverage

### Description

**Figure 2.**
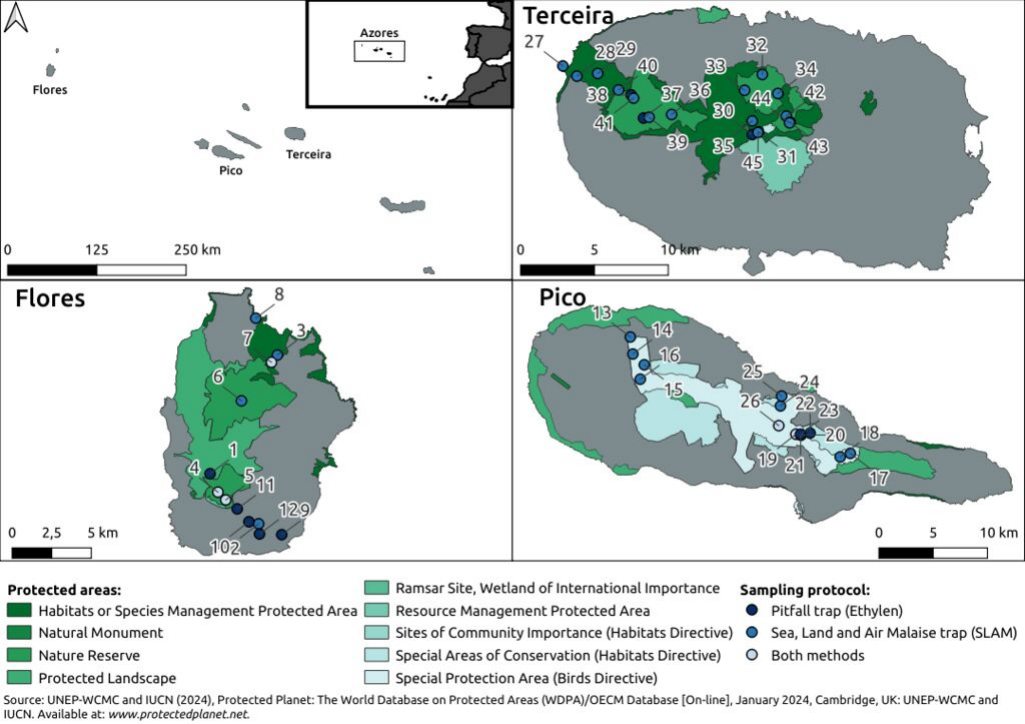
Location of the Azores Archipelago and the sampling sites on islands of Flores, Pico and Terceira. Refer to Table 1 for the description of the sampling sites.

The Azores Archipelago comprises nine volcanic islands located in the Atlantic Ocean between latitudes 37° and 40° N (Fig. 2). The Archipelago spreads over 500 km in a W/NW–E/SE direction. All islands are oceanic of recent volcanic origin and the prevalent climate is temperate, with mild summers and no dry seasons.

### Coordinates

36.844 and 39.690 Latitude; -31.333 and -24.785 Longitude.

## Taxonomic coverage

### Description

The following classes and orders are covered:

- Arachnida: Araneae, Opiliones, Pseudoscorpiones.
- Chilopoda: Geophilomorpha, Lithobiomorpha, Scolopendromorpha, Scutigeromorpha.
- Diplopoda: Chordeumatida, Julida, Polydesmida.
- Insecta: Archaeognatha, Blattodea, Coleoptera, Dermaptera, Ephemeroptera, Hemiptera,Hymenoptera (Formicidae), Lepidoptera, Neuroptera, Orthoptera, Phasmida, Psocodea, Strepsiptera, Thysanoptera, Trichoptera
- Symphyla: Symphyla

## Traits coverage

Additional data on functional traits of Araneae including detailed morphometric measurements for most of the studied species can be accessed in the publication by Macías-Hernández et al. (2020). Trophic preference for all other arthropods are assessed using the publication by Rigal et al. (2018).

## Temporal coverage

### Notes

**Figure 3.**
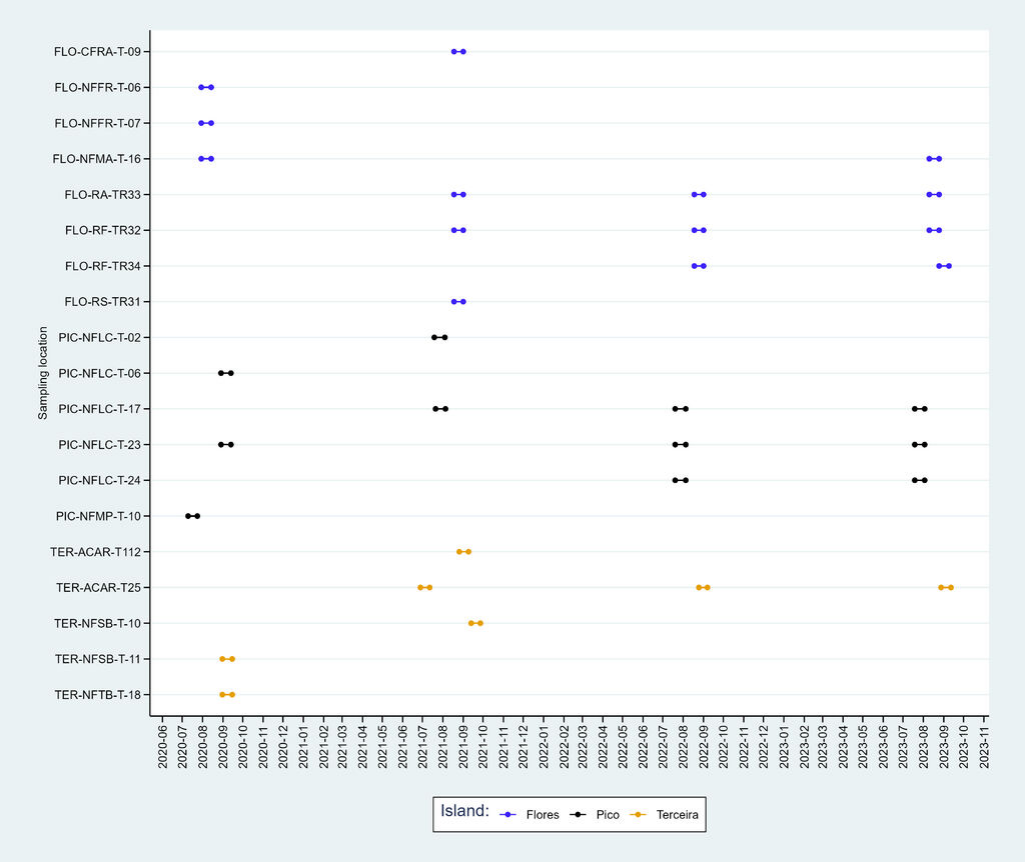
Temporal coverage of the sampling effort using Pitfall traps. Codes of sites as in Table 1. The extremities of the segments refers to the initial and concluding dates of the sampling period.

**Figure 4.**
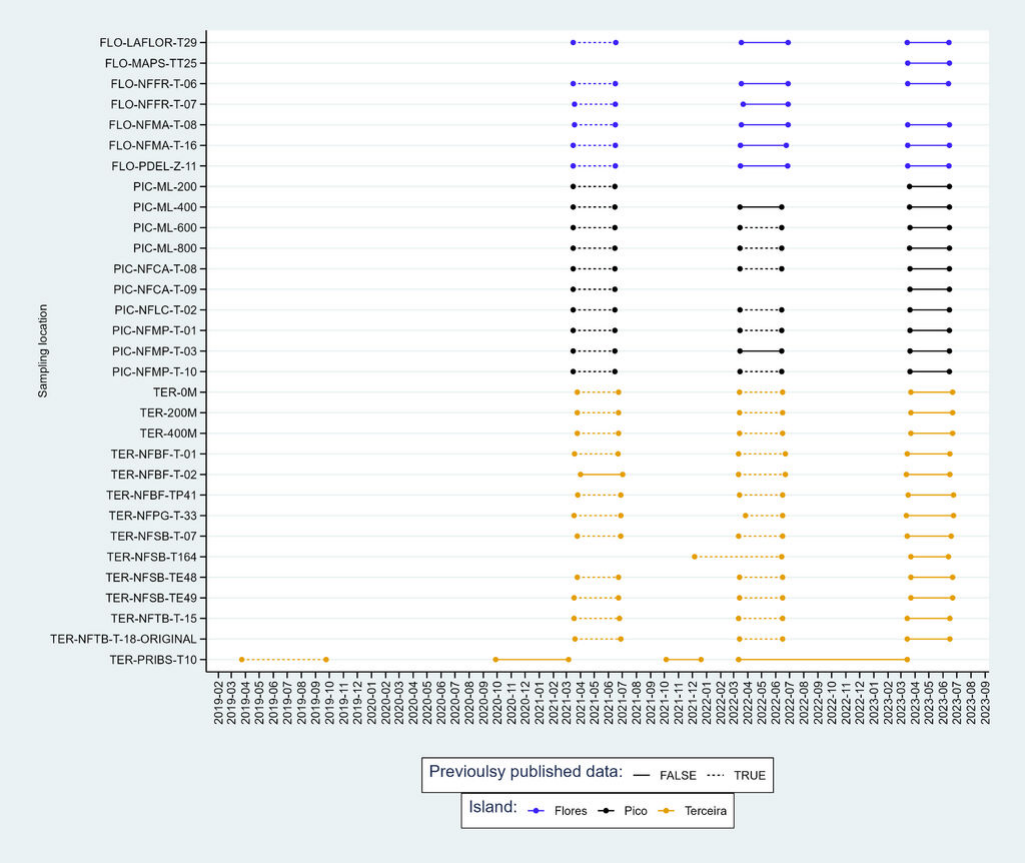
Temporal coverage of the sampling effort using SLAM traps for the current dataset. Previous sampling data are available from Lhoumeau et al. (2022) and Lhoumeau and Borges (2023). Codes of sites as in Table 1. The extremities of the segments refer to the initial and concluding dates of the sampling period. Data that have already been published in Lhoumeau et al. (2022) and used for the analysis of the complete dataset is mentioned in a dashed line.

Temporal graphs (Figs 3, 4) show the range of temporal coverage for all plots and sampling methods used to evaluate the status of the forest plots in which invasive plants were removed and endemic plants planted.

## Collection data

### Collection name

Dalberto Teixeira Pombo

### Collection identifier

DTP

### Specimen preservation method

Ethanol 96%

### Curatorial unit

Curator: Paulo A. V. Borges

## Usage licence

### Usage licence

Creative Commons Public Domain Waiver (CC-Zero)

## Data resources

### Data package title

Monitoring arthropods under the scope of LIFE-BEETLES project – Baseline Data

### Resource link

https://doi.org/10.15468/gp4mfm

### Alternative identifiers

https://www.gbif.org/dataset/72d2dc73-0a10-4e7a-adaa-cab3c440b937; http://ipt.gbif.pt/ipt/resource?r=life_beetles

### Number of data sets

2

### Data set 1.

#### Data set name

Event table

#### Data format

Darwin Core Archive format

#### Character set

UTF-8

#### Download URL

http://ipt.gbif.pt/ipt/resource?r=life_beetles

#### Data format version

Version 1.3

#### Description

The dataset was published in the Global Biodiversity Information Facility platform, GBIF (Borges and Lhoumeau 2024). The following data table includes all the records for which a taxonomic identification of the species was possible. The dataset submitted to GBIF is structured as a sample event dataset that has been published as a Darwin Core Archive (DwCA), which is a standardised format for sharing biodiversity data as a set of one or more data tables. The core data file contains 491 records (eventID). This GBIF IPT (Integrated Publishing Toolkit, Version 2.5.6) archives the data and, thus, serves as the data repository. The data and resource metadata are available for download in the Portuguese GBIF Portal IPT (Borges and Lhoumeau 2024).

**Data set 1.** 

Column label	Column description
id	Unique identification code for sampling event data.
eventID	Identifier of the events, unique for the dataset.
samplingProtocol	The sampling protocol used to capture the species (SLAM or Pitfall).
sampleSizeValue	The numeric amount of time spent in each sampling.
SampleSizeUnit	The unit of the sample size value.
eventDate	Range during which the record was collected.
eventRemarks	In the case of SLAM traps, the verbatim original representation of the date and time information for an Event (season and year). In the case of Pitfall traps, the number of the pitfall trap along the transect.
habitat	The habitat from which the sample was obtained.
locationID	Identifier of the location.
islandGroup	Name of archipelago, always Azores in the dataset.
island	Name of the island.
country	Country of the sampling site, always Portugal in the dataset.
countryCode	ISO code of the country of the sampling site, always PT in the dataset.
stateProvince	Name of the region of the sampling site.
municipality	Municipality of the sampling site.
locality	Name of the locality.
minimumElevationInMetres	The lower limit of the range of elevation (altitude, above sea level), in metres.
locationRemarks	Details on the locality site.
decimalLatitude	Approximate decimal latitude of the trap.
decimalLongitude	Approximate decimal longitude of the trap.
geodeticDatum	The ellipsoid, geodetic datum or spatial reference system (SRS), upon which the geographic coordinates given in decimalLatitude and decimalLongitude are based, always WGS84 in the dataset.
coordinateUncertaintyInMetres	Uncertainty of the coordinates of the centre of the sampling plot.
coordinatePrecision	Precision of the coordinates.
georeferenceSources	A list (concatenated and separated) of maps, gazetteers or other resources used to georeference the Location, described specifically enough to allow anyone in the future to use the same resources.

### Data set 2.

#### Data set name

Occurrence table

#### Data format

Darwin Core Archive Format

#### Character set

UTF-8

#### Download URL

http://ipt.gbif.pt/ipt/resource?r=life_beetles

#### Data format version

Version 1.3

#### Description

The dataset was published in the Global Biodiversity Information Facility platform, GBIF (Borges and Lhoumeau 2024). The following data table includes all the records for which a taxonomic identification of the species was possible. The dataset submitted to GBIF is structured as an occurrence table that has been published as a Darwin Core Archive (DwCA), which is a standardised format for sharing biodiversity data as a set of one or more data tables. The core data file contains 2598 records (occurrenceID). This GBIF IPT (Integrated Publishing Toolkit, Version 2.5.6) archives the data and, thus, serves as the data repository. The data and resource metadata are available for download in the Portuguese GBIF Portal IPT (Borges and Lhoumeau 2024).

**Data set 2.** 

Column label	Column description
id	Unique identification code for species abundance data. Equivalent here to eventID.
type	The nature or genre of the resource, as defined by the Dublin Core standard. In our case "PhysicalObject".
licence	Reference to the licence under which the record is published.
institutionID	The identity of the institution publishing the data.
collectionID	The identity of the collection publishing the data.
institutionCode	The code of the institution publishing the data.
collectionCode	The code of the collection where the specimens are conserved.
datasetName	Name of the dataset.
basisOfRecord	The nature of the data record.
recordedBy	A list (concatenated and separated) of names of people, groups or organisations who performed the sampling in the field.
occurrenceID	Identifier of the record, coded as a global unique identifier.
organismQuantity	A number or enumeration value for the quantity of organisms.
organismQuantityType	The type of quantification system used for the quantity of organisms.
sex	The sex and quantity of the individuals captured.
lifeStage	The life stage of the organisms captured.
establishmentMeans	The process of establishment of the species in the location, using a controlled vocabulary: 'native', 'introduced', 'endemic', 'indeterminate'.
eventID	Identifier of the events, unique for the dataset.
identifiedBy	A list (concatenated and separated) of names of people, groups or organisations who assigned the taxon to the subject.
dateIdentified	The date on which the subject was determined as representing the taxon.
scientificName	Complete scientific name including author and year.
kingdom	Kingdom name.
phylum	Phylum name.
class	Class name.
order	Order name.
family	Family name.
genus	Genus name.
specificEpithet	Specific epithet.
infraspecificEpithet	Infraspecific Epithet.
taxonRank	Lowest taxonomic rank of the record.
scientificNameAuthorship	Name of the author of the lowest taxon rank included in the record.
identificationRemarks	Information about morphospecies identification (code in Dalberto Teixeira PomboCollection).

## Additional information

This comprehensive survey documented a total of 243 arthropod taxa. Of these, 207 taxa were identified at the species or subspecies level, representing four classes, 24 orders and 101 families. Amongst the identified taxa, 46 were endemic, 60 were native non-endemic, 80 were introduced species and 21 remained of undetermined status.

A total of 20,662 individuals were sampled across the study sites. The Hemiptera order was the most prevalent, constituting 11,751 individuals, which accounted for 56.9% of the total sampled arthropods. Within Hemiptera, the Flatidae family was the most abundant, comprising 4021 individuals, equivalent to 19.5% of the total arthropod abundance.

**Figure 5.**
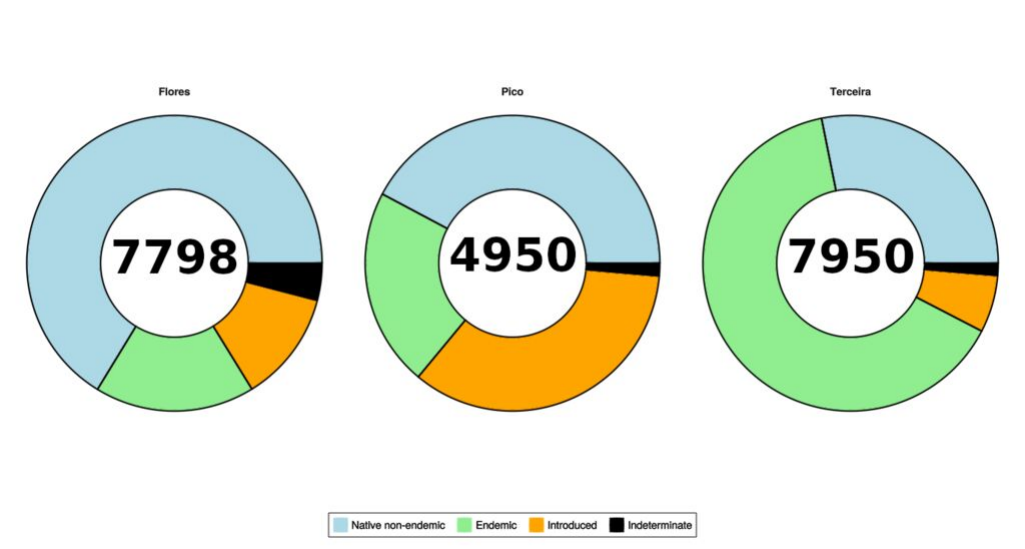
Distribution of arthropod abundance sampled on Flores, Pico and Terceira Islands, categorised by biogeographic origin. Total number of individuals sampled per Island are mentioned within the pie chart. Abundances mentioned are newly sampled and do not include any other databases already published.

The sampled individuals were dominated by native non-endemic species, with 9486 specimens representing 45.8% of the total abundance. Endemic species were well-represented, comprising 7526 individuals or 36.5% of the total sampled community. Introduced species constituted 15.3% of the specimens sampled, with a total count of 3161 individuals. Fig. 5 shows the distribution of sampled arthropod taxa amongst the Islands.

Table 2 presented in this study is a meticulous compilation and comprehensive documentation of arthropod taxa sampled between 2020 and 2023. The LIFE BEETLES project has made a concerted effort to compile the biodiversity of arthropods across various habitats and ecosystems. This Table serves as a comprehensive resource that encapsulates the arthropod diversity within our study areas.

In addition, our analysis was enriched by the integration of previously published data on arthropods sampled using SLAM traps within the same study plots (Lhoumeau and Borges 2023). This combination of datasets provides a rare opportunity to extend not only the breadth of our findings, but also to elucidate temporal trends in arthropod abundance across the sites targeted by the LIFE BEETLES project. By combining these complementary sources of information, we gain a more comprehensive understanding of the dynamics shaping arthropod communities over time. This holistic approach enables us to discern nuanced patterns and fluctuations in arthropod populations, thereby facilitating more informed assessments of ecological changes and conservation needs within our study areas.

The investigation employed the Index of Biotic Integrity (IBI) framework as outlined in the recent publications by Tsafack et al. (2023a) and Tsafack et al. (2023b). This framework provides valuable insights into the ecological status of our study areas and helps identify potential stressors or disturbances affecting arthropod communities over time, as well as any benefits of the conservation actions undergoing during the extent of the LIFE BEETLES project.

**Figure 6.**
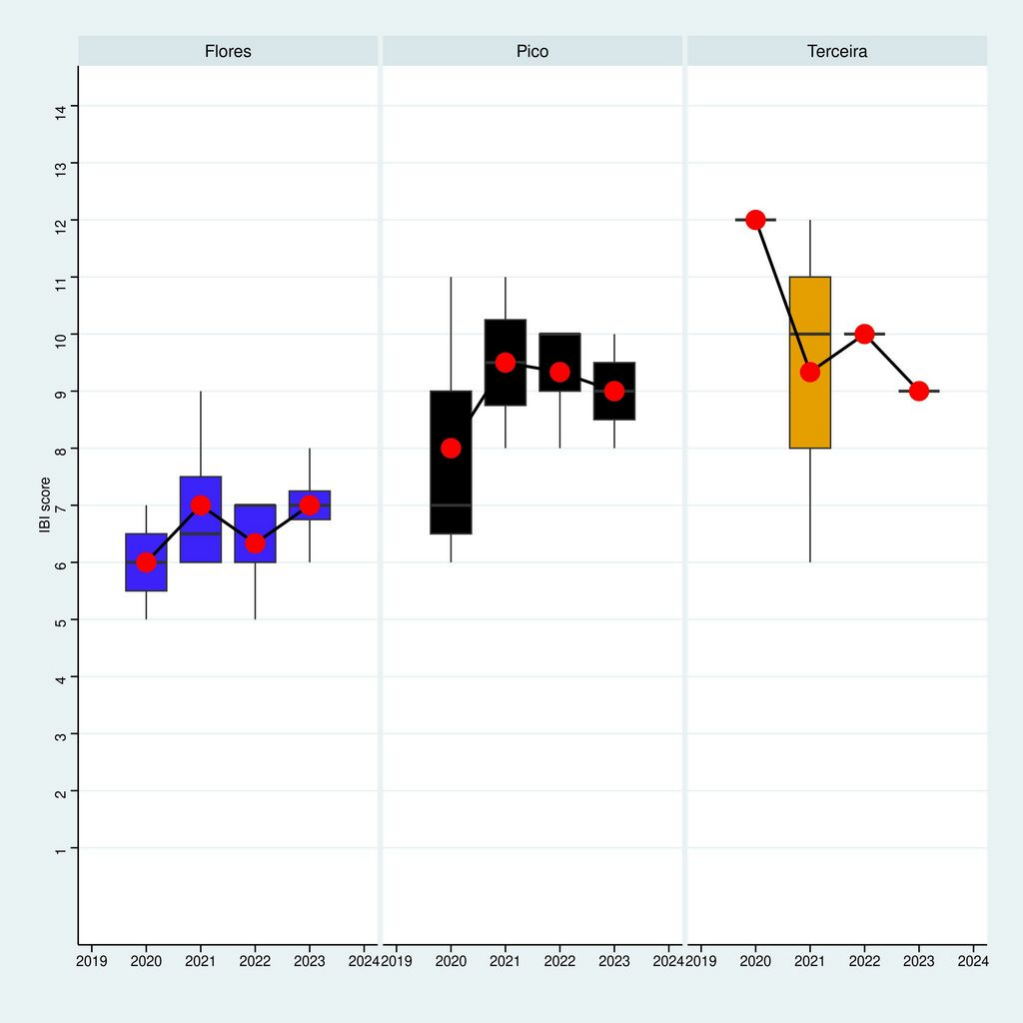
Temporal distribution of the IBI values calculated from data on the epigeal arthropod community sampled by pitfall traps. The mean value for a given island during a given year is represented by the red dots.

**Figure 7.**
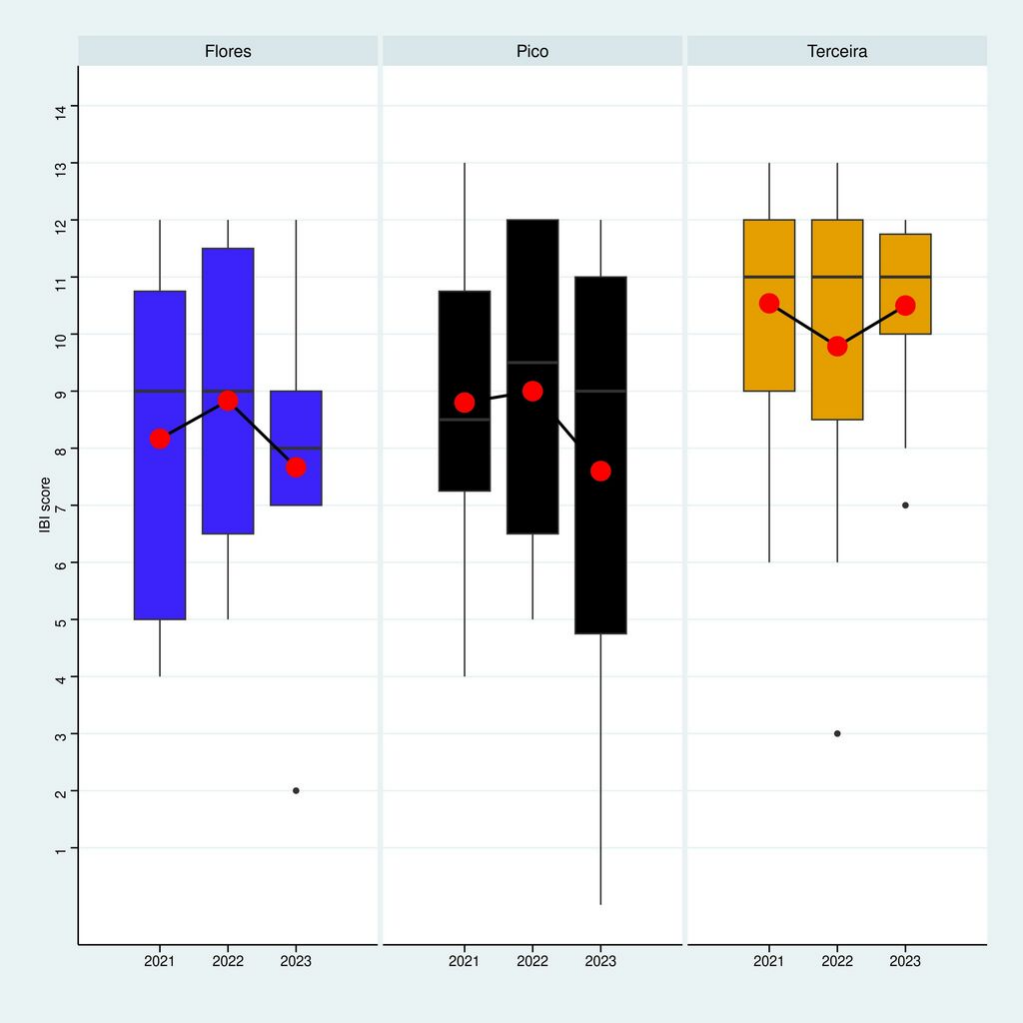
Temporal variation of the IBI value calculated from data on the understorey arthropod community sampled by SLAM traps. The mean value for a given island during a given year is represented by the red dots.

A comparative analysis was conducted by the IBI for two distinct arthropod communities. The ground-dwelling arthropods sampled with pitfall traps and the understorey arthropods sampled with the SLAM traps. This approach enabled the evaluation of two different strata of the ecosystem, with a focus on distinguishing trends amongst islands. Graphs in Figs 6, 7 show the IBI scores for each Island and sampling year.

To assess the temporal evolution of the Index of Biotic Integrity (IBI) for each site, we applied generalised linear mixed models (GLMMs) due to the limited data. We considered the sampling year and the Island as fixed effects and the site as random effect. We used a Poisson distribution for the GLMMs, as it is appropriate for count data like the IBI values. Separate GLMMs were conducted for each sampling method (pitfall and SLAM) to evaluate the changes in IBI over the years in the two different communities.

Terceira statistically exhibits the highest integrity of the epigeal community (see also Fig. 5 that highlights the higher proportion of endemic species on this Island), followed by Pico and Flores. The integrity of the understorey community is also slightly higher, but only marginally significant (p = 0.08). These findings suggest variations in the ecological health and functioning of arthropod communities across the different island ecosystems. Our analysis revealed consistent differences between the ground-dwelling and understorey arthropod communities. The understorey community exhibited higher biotic integrity compared to the ground-dwelling community. However, it should be noted that sampling in the canopy strata is only available for some of these sites within the scope of the BALA project (Pozsgai et al. 2024), so information regarding canopy arthropod communities cannot be evaluated at this stage.

Considering all Islands together, our analysis did not detect any significant temporal variation in biotic integrity within the ground-dwelling arthropod communities across any of the Islands (Year: p > 0.05) (Table 3). This suggests relative stability in the ecological conditions and composition of the ground-dwelling over the sampling periods analysed. However, within the understorey arthropod communities, a significant change was detected (Year: p < 0.05), indicating a very slight decrease in IBI over the years. This decrease appears to be linked to a decline in the Islands of Pico and Flores (see Fig. 7).

Comparing pre- and post-intervention data, we found that the IBI value did not change since the start of the project within the epigean community. On the other hand, we found that the understorey community underwent a change with a global decrease of the biotic integrity. It is likely due to the short period of time considered. Indeed, although an ecosystem can degrade rapidly, its recovery is a much slower process that depends on various factors, including the intensity of the disturbance and the pre-disturbance state of the ecosystem. Therefore, measuring an ecosystem's resilience can be a lengthy process, often requiring long-term monitoring over several years. The duration of this project, which is only a few years, is insufficient to detect any statistically significant changes. Additionally, it is important to note that the time period being considered encompasses not only post-project monitoring, but also the period of action on the study sites (invasive plants removal). The understorey communities appeared to react quicker to the intervention process, likely because of the disturbance generated during the process (plants removal, creation of gaps in the ecosystems, human presence, ...). It is therefore critical to monitor the recovery of the ecosystem after the intervention, while limiting the anthropogenic disturbances.

In conclusion, our study did not detect any immediate changes in the Index of Biotic Integrity (IBI) that could be directly attributed to the conservation actions implemented under the LIFE BEETLES project. However, we remain optimistic about the long-term benefits of improving habitat quality within the intervention areas. Although conservation efforts may not have immediate effects, we anticipate positive changes in arthropod communities, including the targeted species (*Tarphiusfloresensis*, *Pseudanchomenusaptinoides* and *Trechusterrabravensis*), in the near future.

The absence of significant changes in the IBI highlights the complexity and time-lagged nature of ecological responses to conservation interventions. Habitat restoration and enhancement initiatives may take time to produce measurable outcomes, especially in ecosystems with complex ecological dynamics, such as those inhabited by arthropod communities. Therefore, our study provides valuable baseline data and insights into current habitat conditions. However, ongoing monitoring efforts will be crucial for tracking the long-term effectiveness of the LIFE BEETLES project and assessing the trajectory of habitat quality improvement over time.

Furthermore, it is important to acknowledge that the effectiveness of conservation actions goes beyond the immediate outcomes measured by the IBI. Even if there are no detectable changes in habitat quality metrics, the implementation of conservation measures under the LIFE BEETLES project is likely to contribute to broader ecological benefits, such as habitat protection, restoration of ecosystem functions and preservation of biodiversity. These collective efforts are crucial in protecting delicate ecosystems and enhancing the resilience of arthropod communities against ongoing environmental challenges.

Considering these factors, we are dedicated to the objectives of the LIFE BEETLES project and encourage ongoing support and investment in conservation initiatives that aim to improve habitat quality and promote biodiversity conservation. By collaborating with stakeholders, policy-makers and local communities, we can promote a shared commitment to sustainable ecosystem management practices that benefit present and future generations.

## Figures and Tables

**Table 1.** List of the 45 sampled sites in Flores (n = 12), Pico (n = 14) and Terceira (n = 18) Islands. Information about Location ID, sampling method used, decimal coordinates and habitat type are provided.

ID	Site code	Sampling protocol	Longitude	Latitude	Habitat	
1	FLO-CFRA-T-09	Pitfall trap (ethylene glycol)	-31.2299	39.4177	Pasture – Natural	
2	FLO-LAFLOR-T29	Sea, Land and Air Malaise trap (SLAM)	-31.1926	39.3905	Exotic Forest - *Cryptomeria*	
3	FLO-MAPS-TT25	Sea, Land and Air Malaise trap (SLAM)	-31.1846	39.487	Exotic Forest - *Cryptomeria*	
4	FLO-NFFR-T-06	Both methods	-31.2235	39.4074	Native Forest	
5	FLO-NFFR-T-07	Both methods	-31.2175	39.4032	Native Forest	
6	FLO-NFMA-T-08	Sea, Land and Air Malaise trap (SLAM)	-31.2094	39.46	Native Forest	
7	FLO-NFMA-T-16	Both methods	-31.1887	39.4827	Native Forest	
8	FLO-PDEL-Z-11	Sea, Land and Air Malaise trap (SLAM)	-31.2017	39.5074	Mixed Forest	
9	FLO-RA-TR33	Pitfall trap (ethylene glycol)	-31.1753	39.3849	Exotic Forest – Mixed	
10	FLO-RF-TR32	Pitfall trap (ethylene glycol)	-31.1998	39.3914	Exotic Forest – Mixed	
11	FLO-RF-TR34	Pitfall trap (ethylene glycol)	-31.2088	39.3984	Exotic Forest – Mixed	
12	FLO-RS-TR31	Pitfall trap (ethylene glycol)	-31.1916	39.3847	Exotic Forest – Mixed	
13	PIC-ML-200	Sea, Land and Air Malaise trap (SLAM)	-28.4341	38.5348	Mixed Forest	
14	PIC-ML-400	Sea, Land and Air Malaise trap (SLAM)	-28.4311	38.5207	Mixed Forest	
15	PIC-ML-600	Sea, Land and Air Malaise trap (SLAM)	-28.4189	38.5119	Mixed Forest	
16	PIC-ML-800	Sea, Land and Air Malaise trap (SLAM)	-28.4229	38.4999	Mixed Forest	
17	PIC-NFCA-T-08	Sea, Land and Air Malaise trap (SLAM)	-28.2	38.4408	Native Forest	
18	PIC-NFCA-T-09	Sea, Land and Air Malaise trap (SLAM)	-28.2106	38.4377	Native Forest	
19	PIC-NFLC-T-02	Both methods	-28.2577	38.4561	Native Forest	
20	PIC-NFLC-T-06	Pitfall trap (ethylene glycol)	-28.2521	38.4555	Native Forest	
21	PIC-NFLC-T-17	Pitfall trap (ethylene glycol)	-28.2527	38.4555	Native Forest	
22	PIC-NFLC-T-23	Pitfall trap (ethylene glycol)	-28.2427	38.4572	Pasture – Semi-natural	
23	PIC-NFLC-T-24	Pitfall trap (ethylene glycol)	-28.2528	38.4561	Pasture – Semi-natural	
24	PIC-NFMP-T-01	Sea, Land and Air Malaise trap (SLAM)	-28.2744	38.4794	Native Forest	
25	PIC-NFMP-T-03	Sea, Land and Air Malaise trap (SLAM)	-28.2733	38.4876	Native Forest	
26	PIC-NFMP-T-10	Both methods	-28.2759	38.463	Native Forest	
27	TER-0M	Sea, Land and Air Malaise trap (SLAM)	-27.3748	38.7666	Native Forest	
28	TER-200M	Sea, Land and Air Malaise trap (SLAM)	-27.3638	38.7604	Mixed Forest	
29	TER-400M	Sea, Land and Air Malaise trap (SLAM)	-27.3476	38.7621	Mixed Forest	
30	TER-ACAR-T112	Pitfall trap (ethylene glycol)	-27.227	38.7251	Mixed Forest – *Eucalyptus*, *Erica*	
31	TER-ACAR-T25	Pitfall trap (ethylene glycol)	-27.2222	38.7267	Mixed Forest – *Eucalyptus*, *Erica*	
32	TER-NFBF-T-01	Sea, Land and Air Malaise trap (SLAM)	-27.2193	38.7618	Native Forest	
33	TER-NFBF-T-02	Sea, Land and Air Malaise trap (SLAM)	-27.2331	38.7521	Native Forest	
34	TER-NFBF-TP41	Sea, Land and Air Malaise trap (SLAM)	-27.2072	38.7502	Native Forest	
35	TER-NFPG-T-33	Sea, Land and Air Malaise trap (SLAM)	-27.2271	38.7334	Native Forest	
36	TER-NFSB-T-07	Sea, Land and Air Malaise trap (SLAM)	-27.2899	38.7372	Native Forest	
37	TER-NFSB-T-10	Pitfall trap (ethylene glycol)	-27.3118	38.735	Native Forest	
38	TER-NFSB-T-11	Pitfall trap (ethylene glycol)	-27.3215	38.7491	Native Forest	
39	TER-NFSB-T164	Sea, Land and Air Malaise trap (SLAM)	-27.3074	38.7355	Native Forest	
40	TER-NFSB-TE48	Sea, Land and Air Malaise trap (SLAM)	-27.3313	38.7521	Native Forest	
41	TER-NFSB-TE49	Sea, Land and Air Malaise trap (SLAM)	-27.3196	38.7471	Native Forest	
42	TER-NFTB-T-15	Sea, Land and Air Malaise trap (SLAM)	-27.2006	38.7364	Native Forest	
43	TER-NFTB-T-18	Pitfall trap (ethylene glycol)	-27.1976	38.7327	Native Forest	
44	TER-NFTB-T-18-ORIGINAL	Sea, Land and Air Malaise trap (SLAM)	-27.198	38.7323	Native Forest	
45	TER-PRIBS-T10	Sea, Land and Air Malaise trap (SLAM)	-27.2226	38.7264	Mixed Forest – *Eucalyptus*, *Erica*	

**Table 2.** Arthropod abundances specifically sampled for this project. The list includes individuals identified at species-level. Scientific name, colonisation status (CS: I – introduced; N - native non-endemic; E – endemic; NA - indeterminate) and abundance per island. Species with bold names are the species targeted by the LIFE BEETLES. Species and abundances mentioned are newly sampled and do not include any other databases already published.

Class	Order	Species name	Species authorship	CS	Flores	Pico	Terceira
Arachnida	Araneae	*Acorigoneacoreensis*	(Wunderlich, 1992)	E	29	41	33
Arachnida	Araneae	*Agynetadecora*	(O. Pickard-Cambridge, 1871)	I	1	2	0
Arachnida	Araneae	*Agynetadepigmentata*	Wunderlich, 2008	E	2	0	0
Arachnida	Araneae	*Canariphantesacoreensis*	(Wunderlich, 1992)	E	0	4	87
Arachnida	Araneae	*Canariphantesjunipericola*	Crespo & Bosmans, 2014	E	5	0	0
Arachnida	Araneae	*Cheiracanthiumerraticum*	(Walckenaer, 1802)	I	0	0	1
Arachnida	Araneae	*Cheiracanthiumfloresense*	Wunderlich, 2008	E	3	0	0
Arachnida	Araneae	*Cheiracanthiummildei*	L. Koch, 1864	I	26	0	0
Arachnida	Araneae	*Clubionaterrestris*	Westring, 1851	I	216	27	6
Arachnida	Araneae	*Cryptachaeablattea*	(Urquhart, 1886)	I	1	8	56
Arachnida	Araneae	*Dysderacrocata*	C. L. Koch, 1838	I	31	42	48
Arachnida	Araneae	*Erigoneatra*	Blackwall, 1833	I	0	46	1
Arachnida	Araneae	*Erigoneautumnalis*	Emerton, 1882	I	2	0	0
Arachnida	Araneae	*Erigonedentipalpis*	(Wider, 1834)	I	2	3	2
Arachnida	Araneae	*Erofurcata*	(Villers, 1789)	I	3	6	4
Arachnida	Araneae	*Gibbaraneaoccidentalis*	Wunderlich, 1989	E	3	6	47
Arachnida	Araneae	*Lathysdentichelis*	(Simon, 1883)	N	21	1	48
Arachnida	Araneae	*Leucognathaacoreensis*	Wunderlich, 1992	E	4	1	6
Arachnida	Araneae	*Macaroeriscata*	(Blackwall, 1867)	N	1	1	17
Arachnida	Araneae	*Macaroerisdiligens*	(Blackwall, 1867)	N	2	0	0
Arachnida	Araneae	*Mermessusbryantae*	(Ivie & Barrows, 1935)	I	0	2	0
Arachnida	Araneae	*Metellinamerianae*	(Scopoli, 1763)	I	0	0	2
Arachnida	Araneae	*Microlinyphiajohnsoni*	(Blackwall, 1859)	N	0	1	100
Arachnida	Araneae	*Miniciafloresensis*	Wunderlich, 1992	E	0	0	2
Arachnida	Araneae	*Neonacoreensis*	Wunderlich, 2008	E	0	1	1
Arachnida	Araneae	*Nerieneclathrata*	(Sundevall, 1830)	I	2	1	1
Arachnida	Araneae	*Nigmapuella*	(Simon, 1870)	I	1	1	0
Arachnida	Araneae	*Oedothoraxfuscus*	(Blackwall, 1834)	I	0	1119	0
Arachnida	Araneae	*Osteariusmelanopygius*	(O. Pickard-Cambridge, 1880)	I	1	0	0
Arachnida	Araneae	*Pachygnathadegeeri*	Sundevall, 1830	I	0	13	0
Arachnida	Araneae	*Palliduphantesschmitzi*	(Kulczynski, 1899)	N	8	19	7
Arachnida	Araneae	*Parasteatodatepidariorum*	(C. L. Koch, 1841)	I	0	0	2
Arachnida	Araneae	*Pardosaacorensis*	Simon, 1883	E	48	217	25
Arachnida	Araneae	*Pelecopsisparallela*	(Wider, 1834)	I	0	0	1
Arachnida	Araneae	*Pholcommagibbum*	(Westrung, 1851)	I	2	0	0
Arachnida	Araneae	*Pholcusphalangioides*	(Fuesslin, 1775)	I	0	0	1
Arachnida	Araneae	*Pisauraacoreensis*	Wunderlich, 1992	E	4	1	2
Arachnida	Araneae	*Porrhoclubionadecora*	(Blackwall, 1859)	N	2	2	10
Arachnida	Araneae	*Porrhoclubionagenevensis*	(L. Koch, 1866)	I	0	1	0
Arachnida	Araneae	*Porrhommaborgesi*	Wunderlich, 2008	E	0	0	1
Arachnida	Araneae	*Rugathodesacoreensis*	Wunderlich, 1992	E	27	36	101
Arachnida	Araneae	*Salticusmutabilis*	Lucas, 1846	I	6	0	0
Arachnida	Araneae	*Savigniorrhipisacoreensis*	Wunderlich, 1992	E	84	2	74
Arachnida	Araneae	*Tenuiphantesmiguelensis*	(Wunderlich, 1992)	N	13	138	57
Arachnida	Araneae	*Tenuiphantestenuis*	(Blackwall, 1852)	I	33	31	23
Arachnida	Araneae	*Theridionmusivivum*	Schmidt, 1956	N	0	2	1
Arachnida	Araneae	*Walckenaeriagrandis*	(Wunderlich, 1992)	E	0	0	4
Arachnida	Araneae	*Xysticuscor*	Canestrini, 1873	N	2	3	0
Arachnida	Araneae	*Xysticusnubilus*	Simon, 1875	I	1	4	0
Arachnida	Araneae	*Zygiellax-notata*	(Clerck, 1757)	I	0	1	0
Arachnida	Opiliones	*Homalenotuscoriaceus*	(Simon, 1879)	N	74	224	10
Arachnida	Opiliones	*Leiobunumblackwalli*	Meade, 1861	N	56	333	548
Arachnida	Pseudoscorpiones	*Chthoniusischnocheles*	(Hermann, 1804)	I	11	10	25
Arachnida	Pseudoscorpiones	*Ephippiochthoniustetrachelatus*	(Preyssler, 1790)	I	1	1	5
Arachnida	Pseudoscorpiones	*Neobisiummaroccanum*	Beier, 1930	I	63	75	0
Chilopoda	Geophilomorpha	*Geophilustruncorum*	Bergsøe & Meinert, 1866	N	1	2	1
Chilopoda	Geophilomorpha	*Strigamiacrassipes*	(C.L. Koch, 1835)	N	0	2	2
Chilopoda	Lithobiomorpha	*Lithobiuspilicornispilicornis*	Newport, 1844	N	11	21	100
Chilopoda	Scolopendromorpha	*Cryptopshortensis*	(Donovan, 1810)	N	1	0	0
Chilopoda	Scutigeromorpha	*Scutigeracoleoptrata*	(Linnaeus, 1758)	I	1	0	7
Diplopoda	Chordeumatida	*Haplobainosomalusitanum*	Verhoeff, 1900	I	0	0	97
Diplopoda	Julida	*Blaniulusguttulatus*	(Fabricius, 1798)	I	5	0	0
Diplopoda	Julida	*Brachyiuluspusillus*	(Leach, 1814)	I	1	0	0
Diplopoda	Julida	*Cylindroiuluspropinquus*	(Porat, 1870)	I	11	1	3
Diplopoda	Julida	*Nopoiuluskochii*	(Gervais, 1847)	I	3	0	0
Diplopoda	Julida	*Ommatoiulusmoreleti*	(Lucas, 1860)	I	234	187	27
Diplopoda	Julida	*Proteroiulusfuscus*	(Am Stein, 1857)	I	26	0	0
Diplopoda	Polydesmida	*Brachydesmussuperus*	Latzel, 1884	I	0	0	3
Diplopoda	Polydesmida	*Oxidusgracilis*	(C.L. Koch, 1847)	I	30	0	0
Diplopoda	Polydesmida	*Polydesmuscoriaceus*	Porat, 1870	I	58	14	4
Insecta	Archaeognatha	*Diltasaxicola*	(Womersley, 1930)	N	4	38	42
Insecta	Archaeognatha	*Trigoniophthalmusborgesi*	Mendes, Gaju, Bach & Molero, 2000	E	1	0	122
Insecta	Blattodea	*Zethasimonyi*	(Krauss, 1892)	N	25	15	94
Insecta	Coleoptera	*Aleocharabipustulata*	(Linnaeus, 1760)	NA	2	0	0
Insecta	Coleoptera	*Alestrusdolosus*	(Crotch, 1867)	E	10	0	0
Insecta	Coleoptera	*Aloconotasulcifrons*	(Stephens, 1832)	NA	0	0	1
Insecta	Coleoptera	*Anaspisproteus*	Wollaston, 1854	N	14	13	45
Insecta	Coleoptera	*Anisodactylusbinotatus*	(Fabricius, 1787)	I	1	1	0
Insecta	Coleoptera	*Anotylusnitidifrons*	(Wollaston, 1871)	NA	198	7	2
Insecta	Coleoptera	*Anotylusnitidulus*	(Gravenhorst, 1802)	NA	1	0	0
Insecta	Coleoptera	*Aspidapionradiolus*	(Marsham, 1802)	I	0	1	0
Insecta	Coleoptera	*Astenuslyonessius*	(Joy, 1908)	NA	3	6	0
Insecta	Coleoptera	*Athetaaeneicollis*	(Sharp, 1869)	NA	2	0	16
Insecta	Coleoptera	*Athetafungi*	(Gravenhorst, 1806)	NA	0	0	1
Insecta	Coleoptera	*Atlantocisgillerforsi*	Israelson, 1985	E	0	13	0
Insecta	Coleoptera	*Calacallessubcarinatus*	(Israelson, 1984)	E	63	30	12
Insecta	Coleoptera	*Calathuscarvalhoi*	Serrano & Borges, 1986	E	0	0	2
Insecta	Coleoptera	*Carpelimuscorticinus*	(Gravenhorst, 1806)	NA	1	2	1
Insecta	Coleoptera	*Carpophilusfumatus*	Boheman, 1851	I	1	1	0
Insecta	Coleoptera	*Cartoderenodifer*	(Westwood, 1839)	I	0	3	0
Insecta	Coleoptera	*Catopscoracinus*	Kellner, 1846	N	18	0	7
Insecta	Coleoptera	*Cedrorumazoricusazoricus*	Borges & Serrano, 1993	E	0	0	13
Insecta	Coleoptera	*Cephenniumvalidum*	Assing & Meybohm, 2021	NA	81	0	0
Insecta	Coleoptera	*Cercyonhaemorrhoidalis*	(Fabricius, 1775)	I	1	1	1
Insecta	Coleoptera	*Chaetocnemahortensis*	(Fourcroy, 1785)	I	0	0	1
Insecta	Coleoptera	*Chrysolina* sp.		I	0	0	1
Insecta	Coleoptera	*Chrysolinabankii*	(Fabricius, 1775)	N	0	1	2
Insecta	Coleoptera	*Cordaliaobscura*	(Gravenhorst, 1802)	NA	1	0	0
Insecta	Coleoptera	*Crotchiellabrachyptera*	Israelson, 1985	E	0	5	0
Insecta	Coleoptera	*Cryptamorphadesjardinsii*	(Guérin-Méneville, 1844)	I	7	0	1
Insecta	Coleoptera	*Cryptophaguscellaris*	(Scopoli, 1763)	I	0	1	0
Insecta	Coleoptera	*Drouetiusazoricusnitens*	Machado, 2009	E	12	0	0
Insecta	Coleoptera	*Drouetiusborgesiborgesi*	(Machado, 2009)	E	0	0	23
Insecta	Coleoptera	*Epitrixcucumeris*	(Harris, 1851)	I	6	0	0
Insecta	Coleoptera	*Epuraeabiguttata*	(Thunberg, 1784)	I	0	3	0
Insecta	Coleoptera	*Euplectusinfirmus*	Raffray, 1910	NA	0	7	1
Insecta	Coleoptera	*Heteroderesazoricus*	(Tarnier, 1860)	E	3	0	0
Insecta	Coleoptera	*Heteroderesvagus*	Candèze, 1893	I	3	0	0
Insecta	Coleoptera	*Longitarsuskutscherai*	(Rye, 1872)	I	3	15	0
Insecta	Coleoptera	*Notothectadryochares*	(Israelson, 1985)	E	1	0	37
Insecta	Coleoptera	*Ocypusaethiops*	(Waltl, 1835)	NA	0	0	67
Insecta	Coleoptera	*Ocypusolens*	(Müller, 1764)	NA	2	5	0
Insecta	Coleoptera	*Ocysharpaloides*	(Audinet-Serville, 1821)	N	1	0	0
Insecta	Coleoptera	*Orthochaetesinsignis*	(Aubé, 1863)	N	0	0	1
Insecta	Coleoptera	*Otiorhynchuscribricollis*	Gyllenhal, 1834	I	2	1	1
Insecta	Coleoptera	*Otiorhynchusrugosostriatus*	(Goeze, 1777)	I	1	1	1
Insecta	Coleoptera	*Paranchusalbipes*	(Fabricius, 1796)	I	5	8	4
Insecta	Coleoptera	*Phenolialimbatatibialis*	(Boheman, 1851)	I	4	0	0
Insecta	Coleoptera	*Phloeonomuspunctipennis*	Thomson, 1867	NA	0	0	1
Insecta	Coleoptera	*Proteinusatomarius*	Erichson, 1840	NA	0	2	7
Insecta	Coleoptera	***Pseudanchomenusaptinoides***	(Tarnier, 1860)	E	0	17	0
Insecta	Coleoptera	*Pseudoophonusrufipes*	(De Geer, 1774)	I	0	1	0
Insecta	Coleoptera	*Pseudophloeophagustenaxborgesi*	Stüben, 2022	E	34	14	6
Insecta	Coleoptera	*Pseudophloeophagustruncorum*	(Stephens, 1831)	N	2	1	0
Insecta	Coleoptera	*Ptenidiumpusillum*	(Gyllenhal, 1808)	I	0	0	1
Insecta	Coleoptera	*Pterostichusvernalis*	(Panzer, 1796)	I	0	1	0
Insecta	Coleoptera	*Quediuscurtipennis*	Bernhauer, 1908	NA	0	1	0
Insecta	Coleoptera	*Rhopalomesitestardyi*	(Curtis, 1825)	I	1	3	0
Insecta	Coleoptera	*Rugilusorbiculatus*	(Paykull, 1789)	NA	1	15	0
Insecta	Coleoptera	*Scymnusinterruptus*	(Goeze, 1777)	N	0	0	1
Insecta	Coleoptera	*Sepedophiluslusitanicus*	Hammond, 1973	NA	1	1	0
Insecta	Coleoptera	*Sericoderuslateralis*	(Gyllenhal, 1827)	I	0	3	1
Insecta	Coleoptera	*Sitonadiscoideus*	Gyllenhal, 1834	I	0	0	2
Insecta	Coleoptera	*Sphaeridiumbipustulatum*	Fabricius, 1781	I	0	0	2
Insecta	Coleoptera	*Sphenophorusabbreviatus*	(Fabricius, 1787)	I	1	1	0
Insecta	Coleoptera	*Stelidotageminata*	(Say, 1825)	I	59	0	1
Insecta	Coleoptera	*Tachyporuschrysomelinus*	(Linnaeus, 1758)	NA	9	2	1
Insecta	Coleoptera	*Tachyporusnitidulus*	(Fabricius, 1781)	NA	3	16	2
Insecta	Coleoptera	***Tarphiusfloresensis***	Borges & Serrano, 2017	E	24	0	0
Insecta	Coleoptera	*Tarphiusfurtadoi*	Borges & Serrano, 2017	E	0	22	0
Insecta	Coleoptera	***Trechusterrabravensis***	Borges, Serrano & Amorim, 2004	E	0	0	17
Insecta	Coleoptera	*Xantholinuslongiventris*	Heer, 1839	NA	0	1	0
Insecta	Dermaptera	*Euborelliaannulipes*	(Lucas, 1847)	I	10	0	0
Insecta	Dermaptera	*Forficulaauricularia*	Linnaeus, 1758	I	1	3	0
Insecta	Hemiptera	*Acalyptaparvula*	(Fallén, 1807)	N	3	0	0
Insecta	Hemiptera	*Acizziauncatoides*	(Ferris & Klyver, 1932)	I	3	1	1
Insecta	Hemiptera	*Acyrthosiphonpisum*	(Harris, 1776)	N	0	0	1
Insecta	Hemiptera	*Anoscopusalbifrons*	(Linnaeus, 1758)	N	0	59	0
Insecta	Hemiptera	*Anthocorisnemoralis*	(Fabricius, 1794)	N	9	0	1
Insecta	Hemiptera	*Aphrodeshamiltoni*	Quartau & Borges, 2003	E	1	31	42
Insecta	Hemiptera	*Campyloneuravirgula*	(Herrich-Schaeffer, 1835)	N	2	9	1
Insecta	Hemiptera	*Cinarajuniperi*	(De Geer, 1773)	N	1566	170	248
Insecta	Hemiptera	*Cixiusazofloresi*	Remane & Asche, 1979	E	305	0	0
Insecta	Hemiptera	*Cixiusazopifajoazopifajo*	Remane & Asche, 1979	E	0	531	0
Insecta	Hemiptera	*Cixiusazoterceirae*	Remane & Asche, 1979	E	0	0	1089
Insecta	Hemiptera	*Cyphopterumadscendens*	(Herrich-Schäffer, 1835)	N	2915	618	483
Insecta	Hemiptera	*Eupteryxazorica*	Ribaut, 1941	E	0	0	54
Insecta	Hemiptera	*Eupteryxfilicum*	(Newman, 1853)	N	1	0	0
Insecta	Hemiptera	*Geotomuspunctulatus*	(A. Costa, 1847)	N	0	1	0
Insecta	Hemiptera	*Heterotomaplanicornis*	(Pallas, 1772)	N	0	2	0
Insecta	Hemiptera	*Kelisiaribauti*	Wagner, 1938	N	0	8	3
Insecta	Hemiptera	*Kleidocerysericae*	(Horváth, 1909)	N	52	4	45
Insecta	Hemiptera	*Loriculacoleoptrata*	(Fallén, 1807)	N	6	8	2
Insecta	Hemiptera	*Megamelodesquadrimaculatus*	(Signoret, 1865)	N	3	115	4
Insecta	Hemiptera	*Monalocorisfilicis*	(Linnaeus, 1758)	N	2	0	2
Insecta	Hemiptera	*Nabispseudoferusibericus*	Remane, 1962	N	0	0	1
Insecta	Hemiptera	*Nezaraviridula*	(Linnaeus, 1758)	I	1	0	0
Insecta	Hemiptera	*Oriuslaevigatuslaevigatus*	(Fieber, 1860)	N	2	0	1
Insecta	Hemiptera	*Philaenusspumarius*	(Linnaeus, 1758)	I	0	0	1
Insecta	Hemiptera	*Piezodoruslituratus*	(Fabricius, 1794)	N	0	0	1
Insecta	Hemiptera	*Pinalitusoromii*	J. Ribes, 1992	E	24	11	80
Insecta	Hemiptera	*Plinthisusbrevipennis*	(Latreille, 1807)	N	0	0	1
Insecta	Hemiptera	*Rhopalosiphoninuslatysiphon*	(Davidson, 1912)	I	1	6	0
Insecta	Hemiptera	*Scolopostethusdecoratus*	(Hahn, 1833)	N	0	1	0
Insecta	Hemiptera	*Siphantaacuta*	(Walker, 1851)	I	0	2	3
Insecta	Hemiptera	*Strophingiaharteni*	Hodkinson, 1981	E	29	12	73
Insecta	Hemiptera	*Triozalaurisilvae*	Hodkinson, 1990	N	0	23	27
Insecta	Hymenoptera	*Hypoponeraeduardi*	(Forel, 1894)	N	15	0	0
Insecta	Hymenoptera	*Lasiusgrandis*	Forel, 1909	N	266	105	149
Insecta	Hymenoptera	*Monomoriumcarbonarium*	(Smith, 1858)	N	1	0	5
Insecta	Hymenoptera	*Tetramoriumcaespitum*	(Linnaeus, 1758)	N	1	1	0
Insecta	Hymenoptera	*Tetramoriumcaldarium*	(Roger, 1857)	I	0	0	13
Insecta	Lepidoptera	*Argyresthiaatlanticella*	Rebel, 1940	E	1	4	1
Insecta	Lepidoptera	*Ascotisfortunataazorica*	Pinker, 1971	E	0	1	0
Insecta	Lepidoptera	*Cyclophoraazorensis*	(Prout, 1920)	E	0	1	0
Insecta	Lepidoptera	*Mythimnaunipuncta*	(Haworth, 1809)	N	0	1	0
Insecta	Lepidoptera	*Scopariacoecimaculalis*	Warren, 1905	E	0	0	2
Insecta	Neuroptera	*Hemerobiusazoricus*	Tjeder, 1948	E	19	1	21
Insecta	Orthoptera	*Eumodicogryllusbordigalensis*	(Latreille, 1804)	I	0	33	0
Insecta	Orthoptera	*Phaneropteranana*	Fieber, 1853	N	0	0	1
Insecta	Phasmida	*Carausiusmorosus*	(Sinéty, 1901)	I	0	0	1
Insecta	Psocodea	*Atlantopsocusadustus*	(Hagen, 1865)	N	5	2	12
Insecta	Psocodea	*Bertkauialucifuga*	(Rambur, 1842)	N	0	9	0
Insecta	Psocodea	*Ectopsocusbriggsi*	McLachlan, 1899	I	21	3	23
Insecta	Psocodea	*Ectopsocusstrauchi*	Enderlein, 1906	N	2	0	0
Insecta	Psocodea	*Elipsocusazoricus*	Meinander, 1975	E	33	28	87
Insecta	Psocodea	*Elipsocusbrincki*	Badonnel, 1963	E	477	9	97
Insecta	Psocodea	*Trichopsocusclarus*	(Banks, 1908)	N	26	87	20
Insecta	Psocodea	*Valenzuelaburmeisteri*	(Brauer, 1876)	N	0	1	0
Insecta	Psocodea	*Valenzuelaflavidus*	(Stephens, 1836)	N	26	40	86
Insecta	Thysanoptera	*Aeolothripsgloriosus*	Bagnall, 1914	N	0	0	7
Insecta	Thysanoptera	*Anisopilothripsvenustulus*	(Priesner, 1923)	I	2	1	0
Insecta	Thysanoptera	*Ceratothripsericae*	(Haliday, 1836)	N	2	1	4
Insecta	Thysanoptera	*Heliothripshaemorrhoidalis*	(Bouché, 1833)	I	1	0	19
Insecta	Thysanoptera	*Hercinothripsbicinctus*	(Bagnall, 1919)	I	2	0	10
Insecta	Thysanoptera	*Hoplothripscorticis*	(De Geer, 1773)	N	3	9	30
Insecta	Thysanoptera	*Hoplothripsulmi*	(Fabricius, 1781)	I	0	0	12
Insecta	Trichoptera	*Limnephilusatlanticus*	Nybom, 1948	E	1	8	1

**Table 3.** Summary of the generalised linear mixed model fit by Maximum Likelihood (Laplace Approximation) that compares the temporal differences in IBI scores amongst islands during the LIFE BEETLES project.

**Ground-dwelling communities (sampled by pitfall traps)**:				
	Estimate	Standard Error	Z value	p-value (signif.)
Intercept	-12.5	113.60	-0.110	0.91 (NS)
Year	0.007	0.056	0.127	0.90 (NS)
Island - Pico	0.293	0.145	2.03	0.042 (*)
Island - Terceira	0.43	0.16	2.67	0.01 (**)
**Understorey communities (sampled by SLAM traps)**:				
	Estimate	Standard Error	Z value	p-value (signif.)
Intercept	16.3	1.69	9.65	<2e-16 (***)
Year	-0.007	0.0008	-8.48	<2e-16 (***)
Island - Pico	0.019	0.135	0.140	0.8884 (NS)
Island - Terceira	0.218	0.125	1.746	0.0809 (NS)
